# Health state utility estimates for value assessments of novel treatments in Huntington’s disease: a systematic literature review

**DOI:** 10.1186/s12955-024-02242-1

**Published:** 2024-04-16

**Authors:** Ruta Sawant, Kyle Paret, Jennifer Petrillo, Aaron Koenig, Sorrel Wolowacz, Naoko Ronquest, Hugh Rickards

**Affiliations:** 1 Sage Therapeutics, Inc, 215 First Street, Cambridge, MA 02142 USA; 2 Health Economics, RTI Health Solutions, 3040 East Cornwallis Road, Research Triangle Park, Research Triangle Park, NC USA; 3 Health Economics, RTI Health Solutions, The Pavilion, Towers Business Park, Wilmslow Road, Didsbury, Manchester, UK; 4 Institute of Clinical Sciences, College of Medical and Dental Sciences, University of Birmingham, 32-34 Colmore Circus Queensway, Birmingham, UK

**Keywords:** Huntington disease, Neurodegenerative diseases, Systematic review, Quality of life, Quality-adjusted life years, Cost-effectiveness analysis

## Abstract

**Background:**

Huntington’s disease (HD) is a progressive neurodegenerative disease with a devastating impact on patients and their families. Quantifying how treatments affect patient outcomes is critical for informing reimbursement decisions. Many countries mandate a formal value assessment in which the treatment benefit is measured as quality-adjusted life-years, calculated with the use of utility estimates that reflect respondents’ preferences for health states.

**Objective:**

To summarize published health state utility data in HD and identify gaps and uncertainties in the data available that could be used to inform value assessments.

**Methods:**

We conducted a systematic literature review of studies that used preference-based instruments (e.g., EQ-5D and SF-6D) to estimate utility values for people with HD. The studies were published between January 2012 and December 2022.

**Results:**

Of 383 articles screened, 16 articles reported utility values estimated in 11 distinct studies. The utility measure most frequently reported was EQ-5D (9/11 studies). Two studies reported SF-6D data; one used time trade-off methods to value health state descriptions (vignettes). Although utility scores generally worsened to a lower value with increased HD severity, the estimates varied considerably across studies. The EQ-5D index range was 0.89 − 0.72 for mild/prodromal HD and 0.71 − 0.37 for severe/late-stage disease.

**Conclusions:**

This study uncovered high variability in published utility estimates, indicating substantial uncertainty in existing data. Further research is needed to better understand preferences and valuation across all stages and domains of HD symptoms and the degree to which generic utility measures capture the impact of cognitive changes on quality of life.

**Supplementary Information:**

The online version contains supplementary material available at 10.1186/s12955-024-02242-1.

## Introduction

Huntington’s disease (HD) is a hereditary neurodegenerative disease characterized by cognitive and motor decline and behavioral symptoms [1]. Although HD is considered rare, it affects patients worldwide. A recently published meta-analysis estimated the pooled prevalence across studies in Europe, North America, South America, and Asia to be 4.88 per 100,000 people (95% confidence interval, 3.38–7.06) [2]. The impact of HD on patients and their family members is significant [3–5]. According to a cohort study of medical records of primary care patients in the United Kingdom, a significantly higher relative risk of psychotic disorders, depression, insomnia, dementia, weight loss, pneumonia, and falls was observed in patients with HD compared with the demographically matched general population [4]. Despite the well-documented substantial disease burden of HD, the benefits of currently available treatments are limited to the management of motor and psychiatric symptoms. There are several new compounds under investigation that have the potential to improve symptoms or delay progression of symptomatology [6]. Further evidence may be required to ensure approved treatments are reimbursed by payers.

In many countries, determining whether the price of a new treatment is justified by the benefits that it brings to patients and their care partners requires a formal value assessment on the incremental cost per quality-adjusted life-year (QALY) gained [7]. To ensure that patients will have the ability to access innovative treatments if and when they receive market authorization, quantifying the degree to which the new treatment impacts patients’ QALYs is needed. The gain in QALYs with a treatment is estimated by adjusting the value of each year of life according to patients’ health state and quality of life (QOL). Each year of life is weighted with the use of health state utility (HSU) values, where a value of 1 represents full health and 0 represents dead (or a health state equivalent to being dead) [8]. Although health-related quality-of-life (HRQOL) can be measured by both disease-specific instruments (such as the Huntington’s Disease health-related Quality of Life questionnaire [HDQoL]) and generic instruments (such as 36-Item Short-Form Survey [SF-36]), HSU values are recommended to be based on generic measures so that the QALYs can be compared across health conditions. Furthermore, to place higher value on treatments that improve problems that are more important to patients and the general population, payers typically prefer utility values based on preference-based instruments that account for trade-offs among people’s preferences across different dimensions of health attributes. Hence, the value of a life-year is often estimated using generic, preference-based, HRQOL measures, such as the EQ-5D and the short-form 6-dimension (SF-6D), to estimate HSUs and reflect preferences for various health states across a disease continuum [9].

A systematic review by van Lonkhuizen and colleagues [10] summarized studies describing or evaluating self-reported QOL and HRQOL by individuals with genetically or clinically confirmed HD (i.e., premanifest or manifest HD). While that article made a significant contribution toward researchers’ understanding the determinants of a variety of multidimensional QOL outcomes in individuals with premanifest and/or manifest HD, the search strategy focused on identifying general QOL and HRQOL literature rather than studies that report HSU values. As a result, preference-based utility values were available in only 2 of the 30 studies included in their review. To evaluate the availability of published, preference-based utility values associated with different health states in patients with HD and to assess which estimation methods may be most appropriate for HSUs in HD, a review of the existing literature is required. To our knowledge, no article reviewing the existing literature reporting utility values of patients with HD has been published.

The primary objective of this study was to identify published utility data in HD and identify any gaps and/or uncertainty in the data that may suggest caution for use in value assessments or the need for further research. We conducted a systematic literature review to identify studies reporting utility estimates for people with HD and summarized the data for the overall population as well as by stage of disease.

## Materials and methods

### Databases, search strategy, and selection process

A systematic literature review to identify studies reporting utility estimates for people with HD was conducted according to a predefined protocol. An electronic literature search from January 2012 to December 2022 was performed using the electronic medical literature databases Embase, MEDLINE, and Cochrane Library. To identify research abstracts not indexed in medical literature databases, we also included in the search conference abstracts published in the last 2 years (2020–2022) from the Professional Society for Health Economics and Outcomes Research (ISPOR), Huntington Study Group, European Huntington’s Disease Network, and International Society for Quality-of-Life Research.

The inclusion and exclusion criteria (provided in Online Resource 1, Table S1), were based on a strategy to identify the population and disease condition, interventions, comparators, outcomes, and study types of interest). Our search strategy targeted articles and conference abstracts that included utility estimation studies and utility estimates generated as part of economic evaluations for HD. The search string included the key Medical Subject Headings (MeSH) term “Huntington Disease” (and variants) in combination with a variety of key terms pertaining to utility studies (such as “health utility,” “TTO,” “EQ-5D,” “SF-36,” “SF-6D,” “health related quality of life,” “quality adjusted life years”). Details of the full search strategy used in PubMed are provided in Online Resource 1, Table S2; the strategy was adapted for the other database searches. Reference lists of identified review studies were checked for source articles that may have been missed in the primary searches. Articles not published in English, and studies that did not explicitly estimate utility outcomes of patients with HD were excluded.

This review was conducted in accordance with the Preferred Reporting Items for Systematic Reviews and Meta-Analyses (PRISMA) guidelines to ensure transparency in performing and the reporting of the review [11]. Each title and abstract of a study identified from an electronic database or from Internet searches was reviewed by 2 researchers for eligibility according to the inclusion and exclusion criteria. Discrepancies were resolved by consensus. When a consensus was not reached, a third researcher was consulted. Full texts of studies selected were obtained and reviewed by two research team members for eligibility according to the inclusion and exclusion criteria. Each selected study was reviewed by another study member to check for and avoid potential error and bias. Any discrepancies were resolved by consensus; when a consensus was not reached, a third researcher was consulted.

### Outcomes extraction strategy

Once the full set of published studies reporting utility values was identified, evidence tables were created for the following items: title, author(s), publication year, study type, HD severity distribution and method/measure used for severity determination, other population features (e.g., sample size, average age, percentage male vs. female), and key utility outcomes (e.g., measure used, value set applied, respondent type [patient or proxy]) when reported. Utility values were further broken down by HD staging, when available, to capture utility changes throughout disease progression.

Data were extracted from full-text publications, where available. When a single study’s results were reported in multiple publications (i.e., an abstract or poster and a subsequent full-text publication or multiple full-text publications), the final full-text publication with the richest source of data was selected for data extraction. When relevant information (e.g., respondent type, value set applied) was not reported in the full-text journal publication, we searched secondary reports (e.g., abstract, poster, subsequent publications) to seek information that could be reported elsewhere.

## Results

**Fig. 1:**
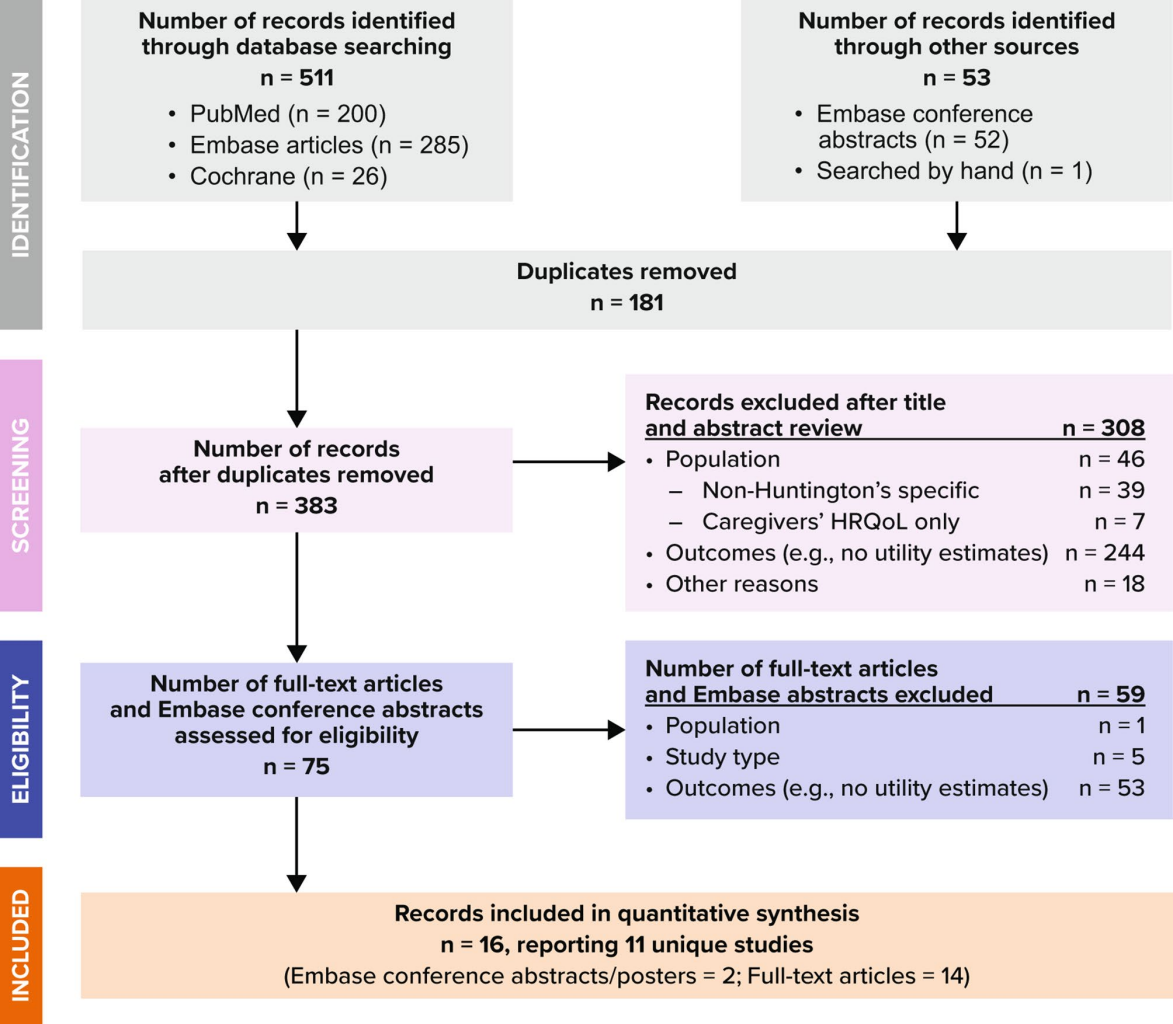
PRISMA diagram for study inclusion and exclusion HRQOL = health-related quality of life, PRISMA = Preferred Reporting Items for Systematic Reviews and Meta-Analyses Adapted from Moher et al. [12]

The inclusion and exclusion processes are shown in the PRISMA flowchart (Fig. 1) [11, 12]. A total of 383 unique publication abstracts, including conference abstracts, were retrieved from database searches. A review of these abstracts identified 66 articles and 9 conference posters that could have met the inclusion criteria for our review. After the full text of the 66 articles were reviewed, 52 were excluded because they did not report utility values for individuals with HD. The final number of articles and conference posters included in this systematic literature review was 16 (14 articles reporting 11 unique studies and 2 conference posters).

Table 1 presents a list of the included studies and key features of each study. Four of the 14 articles [13–16] identified in Fig. 1 reported utility data originating from the same study. Carlozzi et al. [16] was identified as the primary report due to the richness of reported utility data, and the other 3 articles [13–15] were examined for any additional relevant information. In addition, two conference abstracts identified were earlier presentations of data published in subsequent articles by the same authors (Claassen et al. [17], Rodriguez Santana et al. [18]). Therefore, 11 distinct studies from the 16 identified publications were included in our analysis. Table 1 lists the characteristics of the 11 studies. Among the 11 distinct studies, 4 included participants located in the US, 2 in the UK, and 3 were multinational (2 including European countries and 1 including both US and European countries). The remaining 2 studies included participants located in Spain and Canada.

**Table 1:** Selected studies and key study features

Author (year)	Country	Study design	Study populations	HRQOL measures included
Calvert et al. [25] (2013)	UK	▪ Cross-sectional survey ▪ Recruited through disease charities, specialist neurology clinics at University Hospital Birmingham Trust and via the UK Clinical Research Networks	▪ Patients with HD (whether self-reported or confirmed diagnosis was not reported) ▪ Mean age: 57.1 years ▪ Male: 45.1% ▪ Mean no. of years since diagnosis: 4.9	Utility measure: EQ-5D index score (3L vs. 5L versions of EQ-5D not reported, value set not reported)
Carlozzi et al. [27] (2014)	US	▪ Cross-sectional surveys of patients with HD and their proxies (caregivers) ▪ Recruited through online panel and a display at 2012 HDSA annual meeting ▪ Patients’ characteristics (e.g., diagnoses, gene testing, years since diagnosis) and outcomes were self-reported	▪ Individuals with HD (self-reported) (*n* = 132) ▪ Caregivers (*n* = 40) ▪ Mean age: 40.8 years ▪ Female: 48% ▪ Mean no. of years since diagnosis: 4.8	Utility measure: EQ-5D index score (3L vs. 5L versions of EQ-5D not reported, value set not reported) Other measures: ▪ HD-PRO-TRIAD ▪ Neuro-QOL
Carlozzi et al. [16] (2016) Other articles based on the same study: Carlozzi et al. [13] (2015), Carlozzi et al. [14] (2018), Carlozzi et al. [15] (2019)	US	▪ Longitudinal, prospective, observational study ▪ Clinician-administered data collection (for clinical and demographic outcomes) ▪ Patient-reported assessments completed by patients (or with assistance from a family member or site staff) ▪ Recruitment from specialized treatment centers, other ongoing studies (e.g., Predict-HD), and the National Huntington Disease Roster and existing online medical record data capture systems	▪ Adults (aged ≥ 18 years) with prodromal and/or manifest HD, positive CAG test for gene expansion and/or clinical diagnosis (*n* = 536) ▪ Majority of prodromal patients were from the Predict-HD study ▪ Mean age: 48.74 years ▪ Female: 59.0% ▪ Mean (SD) no. of years since diagnosis (for nonprodromal patients): 3.97 (4.22)	Utility measure: EQ-5D index (3L vs. 5L versions of EQ-5D not reported, value set not reported) Other measures: ▪ HDQLIFE, SF (new 4-item scale), PROMIS, Neuro-QOL ▪ WHODAS 2.0, RAND-12
Claassen et al. [17] (2022) Claassen et al. [47] (2021), abstract	US	▪ One-time computer-assisted phone interviews to estimate utilities for 4 severity levels of HD chorea described by vignettes	▪ Participants (*n* = 155) were general US public recruited by an online panel ▪ Mean age: 47 years ▪ Male: 48.4%	▪ Other measures: ▪ TTO ▪ EQ-5D VAS (normalized 0 to 1)
Dorey et al. [19] (2016)	Spain	▪ European HD burden survey (Euro-HDB) ▪ Cross-sectional survey	▪ Patients with a well-established diagnosis of HD and their caregivers ▪ Recruited with the help of a hospital neurologist ▪ Mean age: 49.66 years ▪ Male: 49.09% ▪ Derived mean no. of years since diagnosis: 5.54	Utility measure: EQ-5D-3L (value set not reported) ▪ Other measure included: Huntington Quality of Life Instrument (H-QoL-I)
Exuzides et al. [21] (2022)	US	▪ A cross-sectional study based on survey data ▪ Primary data: a prospective, customized survey administered by the Rare Patient Voice (July 2019–August 2019) ▪ Control: a nationally representative online survey, National Health and Wellness Survey ▪ Patients’ characteristics and outcomes were self-reported ▪ HD patients and care partners reported their “own” HRQOL level (i.e., no proxy rating)	▪ Individuals with HD (self-reported diagnosis) between ages 18–70 years (*n* = 41; mean age: 45.61 years; 68.3% female) ▪ Matched general population (*n* = 123; mean age: 45.61 years; 68.3% female)	Utility measures: ▪ EQ-5D-5L Index (value set not reported) ▪ EQ-5D VAS Other measure: PHQ-9
Hawton et al. [23] (2019)	12 European countries	▪ A longitudinal, observational study from the European Huntington’s Disease Network (the REGISTRY study) ▪ Annual evaluations of demographic, clinical, and patient-reported outcomes conducted by the investigators ▪ Between annual visits, every 2 months, participants completed surveys on demographic, clinical, and patient-reported outcome measures	▪ Patients with HD in the REGISTRY study were clinically diagnosed ▪ Mean age: 48.6 years ▪ Female: 53% ▪ Mean duration since diagnosis: 4.5 years	▪ Utility measure: SF-6D ▪ Participant responses to SF-36 were converted to SF-6D–based utility values [34]
Hocaoglu et al. [26] (2012)	UK	▪ Prospective cross-sectional survey ▪ HD patients recruited by mail-out via the HD association ▪ Subset of HRQOL survey ▪ Patient characteristics and outcomes were self-reported	▪ Persons with HD (*n* = 105) ▪ Self-reported diagnosis (87% reported having positive gene status, 67% reported having clinical diagnosis) ▪ Female: 58% (61/105) ▪ Mean age: 56.42 years	Utility measures: ▪ EQ-5D index (3L vs. 5L versions of EQ-5D not reported, value set not reported) ▪ EQ-5D VAS Other measures: ▪ SF-12v2 ▪ HDQoL
Quinn et al. [20] (2016)	UK Netherlands Germany Norway	▪ A single-blind, multi-center, randomized controlled trial to demonstrate the efficacy of a 12-week exercise program for patients with HD ▪ Trial sites = the ENROLL-HD/Registry sites ▪ All assessments were collected at the trial sites	▪ Patients with genetically confirmed diagnosis of HD ▪ Patients receiving routine HD clinical care or attending the ENROLL-HD study ▪ Control group (*n* = 15, males/females = 7/8, mean age: 51 years) ▪ Intervention group (*n* = 17, males/females = 9/8, mean age: 53 years)	Utility Measure: EQ-5D-3L (value set not reported)
Rodriguez Santana et al. [18] (2022), Rodriguez Santana et al. [48] (2022), abstract	Germany, France, Italy, Spain, UK, US	▪ A retrospective analysis of the Huntington’s Disease Burden of Illness Study (HDBOI) cross-sectional dataset ▪ Demographic, clinical, and health care resource utilization reported by treating physicians ▪ HRQOL, nonmedical and indirect costs reported by patients and caregivers	▪ *N* = 336 patients with HD (8% [*n* = 27] by proxy for participants with a severe cognitive deficit, self-reported for the remainder of the sample) ▪ Mean age: 47.3 years ▪ Female: 35%	Utility Measures: ▪ EQ-5D-5L with England value set ▪ SF-6D
Shaw et al. [22] (2022)	Canada	▪ Cross-sectional online survey ▪ 3 types of participants: patients with HD, patient proxies (for patients who were unable to complete the survey), care partners ▪ Patient characteristics and outcomes were self- (or proxy-) reported	▪ 62 adult patients with self-reported diagnosis of HD ▪ A separate set of self-reported unpaid care partners identified as proxies of HD patients ▪ Mean age: 51.2 years ▪ Female: 28% (17/61) Mean (SD) years since diagnosis: 9.9 (7.3)	Utility Measure: EQ-5D, mapped from the SF-36 (UK TTO value set)

CAG = coronary angiogram; HD = Huntington’s disease; HDQoL = Huntington’s Disease health-related Quality of Life questionnaire; HDSA = Huntington’s Disease Society of America; HRQOL = health-related quality of life; PHQ-9 = Patient Health Questionnaire-9; PROMIS = Patient-Reported Outcomes Measurement Information System; SF-36 = 36-Item Short-Form Survey; SF-6D = short-form 6-dimension; SD = standard deviation; TTO = time trade-off; UK = United Kingdom; US = United States; VAS = visual analogue scale; WHODAS 2.0 = World Health Organization Disability Assessment Schedule 2.0

Note: Country refers to location of residence of study participants

The utility measure used most frequently was the EQ-5D index (9 of 11 studies, Table 1). Two studies used the 3-level version of the instrument [19, 20], 2 studies used the 5-level version [18, 21], 1 study mapped the EQ-5D from SF-36 data [22], and the remaining 4 studies did not report the EQ-5D version. Two studies used the SF-6D to estimate utility by disease stage in patients with HD [18, 23], one of which also reported EQ-5D utility values [18]. One study did not report EQ-5D or SF-6D but instead estimated utility values associated with 4 levels of chorea severity described in vignettes using time trade-off methods in a sample of the general population [17].

A variety of disease-specific HRQOL measures were used in the studies alongside preference-based generic utility measures, including the HD-PRO-TRIAD, Quality of Life in Neurological Disorders, HDQLIFE Short Form, the Huntington Quality of Life Instrument (H-QoL-I), and the HDQoL. Also, EQ-5D visual analogue scale (VAS) scores were often reported alongside the EQ-5D index scores. For example, a cross-sectional survey study by Exuzides and colleagues [21] reported both the EQ-5D index and VAS scores.

The demographic and clinical characteristics of the populations included in the 10 studies that included patients with HD are reported in Table 1 (the 11th study, Claassen et al. [17], was a vignette valuation study in the general population). The study populations appear to be generally representative of the overall HD population, with a similar number of men and women [1]. However, we identified 3 cross-sectional surveys with more unbalanced samples (Exuzides et al. [21] with 68.3% female; Shaw et al. [22] with 28% female; Rodriguez Santana et al. [18] with 35% female). The average age of the participants was between 40 and 58 years, which appears to be consistent with other studies in HD that describe age at onset (mean = 30–50 years; range = 2–85) and disease duration (mean = 17–20 years) [24]. The verification of diagnosis differed somewhat among the studies (Table 1), with studies conducted at clinical sites using clinical diagnosis [16, 18, 23] or a positive gene status [20]. Two studies did not report details on how diagnoses were verified [19, 25], and 4 studies allowed participant self-reported diagnoses, either by genetic identification or clinical diagnosis [21, 22, 26, 27]. Only one of these studies [26] reported the proportion of patients in which the diagnosis was based on gene mutation status or clinical diagnosis: 87% of participants reported a positive gene mutation status and 67% reported clinical diagnosis. Where reported, the mean number of years since diagnosis varied from 3.97 (Carlozzi et al. [16]) to 9.0 years (Shaw et al. [22]); in 4 studies, it was not reported.

Among the 11 studies, 2 were based on site-administered surveys along with clinical observations [16, 23], and 5 were cross-sectional self-administered surveys [21, 22, 25–27]. Two studies were based on cross-sectional survey data collected from patients and physicians in an existing HD dataset (Rodriguez Santana et al. [18]; Huntington’s Disease Burden of Illness Study, Dorey et al. [19]; European-Huntington’s disease burden study). The remaining 2 studies included a vignette study in the general population [17] and a randomized controlled trial [20].

Among the surveys of patients with HD, more than half (7/9) were cross-sectional studies that relied on self-/proxy- or physician survey–reported outcomes completely; the remaining 2 studies [16, 23] were longitudinal, prospective, observational studies that required clinicians to collect at least some of the key clinical outcomes, such as clinical diagnoses of HD, years since HD diagnosis, staging, and severity. All 9 survey-based studies collected self-reported outcomes from patients with HD; 1 of these studies also collected proxy-rated utility values for all participants [27]. One study collected proxy-rated utility values only for patients who could not respond due to advanced disease [18].

Table 2 summarizes the utility estimates reported in each of the 11 studies identified. Among the 9 studies reporting EQ-5D utility index data, utility estimates for the overall HD population ranged from 0.81 (mean age = 48.74 years, 41.0% male, mean years since diagnosis = 3.97, prodromal HD 38.5%, early HD 38.0%, late HD 23.5%) [16] to 0.3 (mean age = 57.1 years, 45.1% male, mean years since diagnosis = 4.9) [25]. In early-stage and mid-stage disease, the anxiety and depression dimension was the main driver of poor EQ-5D utility scores, while in patients with advanced-stage disease, the main drivers were mobility, followed by the self-care and usual activities [18]. One study used the EQ-5D utility index to compare HD patients with an age- and sex-matched general population cohort [21]. In the cross-sectional survey conducted by Exuzides and colleagues in the US, the mean EQ-5D-5 L index score was significantly lower for patients with HD than for the general population (0.66 vs. 0.81; *P* < 0.001); almost half of the HD group had early-stage disease.

**Table 2:** Selected studies’ key study design features, population characteristics, and utility estimates

Author (Year, country)	Study design	HD severity	Other clinical characteristics	Utility / disutility estimate (health state descriptions, if applicable)
Calvert et al. [25] (2013, UK)	Cross-sectional survey of patients with rare, long-term neurological conditions	Not reported	Mean no. of years since diagnosis: 4.9	▪ EQ-5D index score, mean (95% confidence interval) • Overall: 0.30 (0.41 − 0.19), *n* = 22 Note: patients were allowed to receive “some help” by carers in responding to questions, but no proxy-reported utility scores were reported
Carlozzi et al. [27] (2014, US)	A cross-sectional survey of patients with HD and their proxies (caregivers)	Staging not reported	▪ Mean (SD) years since diagnosis: 4.8 (3.9) ▪ Mean (SD) years with motor symptoms: 5.7 (5.6) ▪ Mean (SD) years with any symptoms: 5.0 (3.5) (caregivers report: 5.7 [4.3]) ▪ Mean (SD) TFC: 6.8 (4.3) ▪ Mean (SD) UHDRS IS: 3.5 (2.3)	▪ EQ-5D index score, mean (SD) • Self: 0.6 (0.3), *n* = 132 • Proxy: 0.6 (0.3), *n* = 40 Note: the mean proxy-reported TFC (5.4) was lower than the mean self-reported mean TFC (6.8)
Carlozzi et al. [16] (2016, US)	Prospective, observational study (self-reported only)	▪ Prodromal HD (TFC 13), *n* = 205 (38.5%) ▪ Early-stage HD (TFC 7–13), *n* = 202 (38.0%) ▪ Late-stage HD (TFC 0–6), *n* = 125 (23.5%) ▪ TFC = clinician rated	▪ Mean (SD) no. of years since diagnosis: 3.97 (4.22) • Early-stage HD: 3.07 (3.71) • Late-stage HD: 5.88 (4.62) ▪ Mean (SD) UHDRS IS score: • All: 84.30 (16.62) • Prodromal HD: 97.71 (5.95) • Early-stage HD: 85.02 (9.65) • Late-stage HD: 61.40 (12.13)	▪ EQ-5D index score, mean (SD) • Prodromal HD: 0.89 (0.12): *n* = 205 • Early-stage HD (TFC: 13 − 7): 0.80 (0.14); *n* = 202 • Late-stage HD (TFC: 6 − 0): 0.71 (0.17); *n* = 125 • All: 0.81 (0.15) ▪ Prodromal HD: positive CAG test for gene expansion but no symptoms
Claassen et al. [17] (2022, US)	Vignettes (health state descriptions) valued by members of the US general population using time trade-off methods	Vignette descriptions of chorea severity: (mild, mild/moderate, moderate/severe, severe)	NA	▪ TTO score, mean (SD) • Mild chorea, 0.64 (0.41) • Mild/moderate chorea, 0.48 (0.47) • Moderate/severe chorea, 0.26 (0.50) • Severe chorea, 0.07 (0.52) ▪ VAS (divided by 100): all, EQ-5D VAS: 0.79 (0.16) • Mild chorea, 0.59 (0.20) • Mild/moderate chorea, 0.47 (0.20) • Moderate/severe chorea, 0.32 (0.19) • Severe chorea, 0.19 (0.17)
Dorey et al. [19] (2016, Spain)	Cross-sectional self-reported survey	Overall severity level distribution was not reported, but the study included patients with low to high severity levels (UHDRS IS score of 10–60,70–80, 90–100)	Mean no. of years since diagnosis: 5.54 Mean functional score (0–7) in Huntington clinical self-reported instrument (H-CSRI): 3.06 (2.53)	▪ EQ-5D-3L index score, mean (SD) • Overall: 0.54 (0.43), *n* = 55 • Low independence; UHDRS IS score ≤ 60 (high level of severity): 0.25 (mean) • High independence; UHDRS IS score > 80 (moderate or better severity): 0.84 (mean)
Exuzides et al. [21] (2022, US)	A cross-sectional study based on survey data	▪ HD patients • Early-stage: 48.8% • Mid-stage: 39.0% • Late-stage: 12.2% Staging definition not reported	HD care partners were recruited but reported their “own” HRQOL level (i.e., no proxy rating)	▪ EQ-5D-5L, mean (SD) • All HD patients: 0.66 (0.21), *n* = 41 • Matched general population for patients: 0.81 (0.17) • HD caregivers: 0.82 • Matched general population for caregivers: 0.84 ▪ EQ-5D VAS, mean (SD) • All HD patients: 58.83 (23.43) • Matched general population: 75.68 (21.00) • HD caregivers: 73.71 • Matched general population for caregivers: 77.32
Hawton et al. [23] (2019, Europe)	A longitudinal, observational study	▪ TFC 13 − 11, *n* = 2,869 ▪ TFC 10 − 7, *n* = 1,755 ▪ TFC 6 − 3, *n* = 1,500 ▪ TFC 2 − 0, *n* = 774 TFC = patient rated	▪ Years since diagnosis • < 1, *n* = 1,323 • 1–4, *n* = 2,255 • 5–9, *n* = 1,388 • ≥ 10, *n* = 449 ▪ Mean years since diagnosis: 4.5	▪ SF-6D utilities by stage, mean (SD) • TFC 13 − 11: 0.767 (0.131), *n* = 4,991 • TFC 10 − 7: 0.675 (0.128), *n* = 2,753 • TFC 6 − 3: 0.633 (0.121), *n* = 2,282 • TFC 2 − 0: 0.575 (0.118) *n* = 774 ▪ SF-6D utilities by years since diagnosis, mean (SD) • < 1: 0.691 (0.140) • 1–4: 0.683 (0.135) • 5–9: 0.659 (0.133) • ≥ 10: 0.640 (0.128)
Hocaoglu et al. [26] (2012, UK)	Prospective survey study	▪ At risk, *n* = 10 ▪ Gene positive, *n* = 17 ▪ Stages • Early, *n* = 9 • Moderate, *n* = 18 • Advanced, *n* = 50	NA	▪ EQ-5D index utilities, mean (SD) • Self-report: 0.56 (0.35), *n* = 105 ▪ EQ-5D VAS scores, mean (SD) • Self-report: 58.38 (23.2)
Quinn et al. [20] (2016, searched by hand, Europe)	Randomized controlled trial evaluating the benefits of exercise in patients with HD	▪ TFC, mean (SD) • 9 (3) in control arm • 8 (3) in active arm	▪ UHDRS TMS, mean (SD) • 32 (14) (control) • 39 (22) (active) ▪ UHDRS SDMT, mean (SD) • 28 (10) (control) • 23 (9) (active)	▪ EQ-5D-3L index at baseline, mean (SD) • Control, index: 0.74 (0.17), *n* = 15 • Intervention, index: 0.77 (0.19) ▪ EQ-5D index postintervention (13 weeks of exercise), mean (SD) • Control, index: 0.75 (0.19) • Intervention, index: 0.81 (0.14)
Rodriguez Santana et al. [18] (2022, Europe and US)	Retrospective analysis of the HDBOI dataset	▪ *n* = 336 for EQ-5D respondents • Early stage (TFC 13 − 7): 38% • Mid stage (TFC 6 − 4): 35% • Advanced stage (TFC 3 − 0): 26% TFC was clinician rated ▪ *N* = 482 for SF-6D respondents	▪ Clinically diagnosed with symptomatic motor HD disease ≥ 12 months before study recruitment	▪ EQ-5D-5L utility estimates (all participants / self-reported / proxy reported), mean (SD) • Early-stage HD: 0.72 (0.22) / 0.74 (0.19) / 0.34 (0.31), *n* = 129/122/7 • Mid-stage HD: 0.62 (0.18) / 0.62 (0.18) / 0.66 (0.26), *n* = 119/115/4 • Advanced-stage HD: 0.37 (0.30) / 0.42 (0.27) / 0.13 (0.31), *n* = 88/72/16 ▪ SF-6D utility estimates (all participants / self-reported / proxy reported), mean (SD) • Early-stage: 0.61 (0.12) / 0.61 (0.11) / 0.54 (0.11) • Mid-stage: 0.56 (0.07) / 0.56 (0.07) / 0.55 (0.06) • Advanced-stage: 0.50 (0.08) / 0.51 (0.08) / 0.44 (0.09) ▪ Staging based on Wild and Tabrizi [36] descriptors • Early-stage: TFC 13 − 7 • Moderate-stage: TFC 6 − 4 • Advanced-stage: TFC 3 − 0
Shaw et al. [22] (2022, Canada)	A cross-sectional online survey	▪ TFC 13 − 11, *n* = 22 ▪ TFC 7–10, *n* = 13 ▪ TFC 6 − 3, n = < 10 ▪ TFC 2 − 0, n = < 10 ▪ No score, *n* = 10	▪ Mean (SD) years since diagnosis: 9.9 (7.3) ▪ 42.4% experienced motor symptom onset	▪ EQ-5D mapped from SF-36 (UK TTO value set), all participants (*n* = 48) • Mean (SD): 0.72 (0.24), *n* = 48 • Median: 0.77

CAG = coronary angiogram; HD = Huntington’s disease; HDBOI = Huntington’s Disease Burden of Illness Study; IS = Independence Scale; NA = not applicable; SD = standard deviation; SDMT = Symbol Digit Modalities Test; SF-36 = 36-Item Short-Form Survey; SF-6D = short-form 6-dimension; TFC = Total Functional Capacity sum of score; TMS = Total Motor Score; TTO = time trade-off; UHDRS = Unified Huntington’s Disease Rating Scale; UK = United Kingdom; US = United States; VAS = visual analogue scale

Note: Country refers to location of residence of study participants

Where data were reported for different levels of severity of HD, utility scores generally worsened with increasing HD severity. In the cross-sectional US Huntington’s Disease Burden of Illness Study, Rodriguez Santana and colleagues [18] reported a decline in the mean (standard deviation [SD]) EQ-5D-5 L index score for patients with advancing disease (early-stage: 0.72 [0.22]; mid-stage: 0.62 [0.18]; and advanced-stage: 0.37 [0.30]). Carlozzi et al. [16] and Dorey et al. [19] also reported decreasing EQ-5D index scores with advancing disease.

Although all studies exploring EQ-5D values for different disease stages observed lower utility values with advancing disease, the score ranges within each disease stage varied considerably across studies. For example, in the cross-sectional study of patients with HD by Rodriguez Santana et al. [18], the mean (SD) EQ-5D index score for patients with early- (Total Functional Capacity sum of score [TFC] 13 − 7), mid- (TFC 6 − 4), or advanced-stage (TFC 3 − 0) HD, as defined by Wild et al. [28], was reported to be 0.72 (0.22), 0.62 (0.18), and 0.37 (0.30), respectively. However, Carlozzi and colleagues’ [16] longitudinal observational study reported the mean (SD) EQ-5D utility index scores for patients in prodromal, early (TFC 13 − 7), and late (TFC 6 − 0) stages of HD to be 0.89 (0.12), 0.80 (0.14), and 0.71 (0.17), respectively. The utility estimate for TFC 13 − 7 was higher in Carlozzi and colleagues (0.80 versus 0.72), and the estimate for TFC 6 − 0 in Carlozzi and colleagues was higher than the estimate for TFC 6 − 4 in Rodriguez Santana (0.71 versus 0.62), despite the latter excluding later stage patients (TFC 3 − 0) where utility was lower (0.37). Both were large studies (336 and 536 patients, respectively, with sample sizes ranging from 88 to 205 within each severity category), with clinician-verified diagnosis and similar mean age (47.3 vs. 48.74 years). There were fewer women in the Rodriguez Santana sample (35% vs. 59% in the Carlozzi sample). Some of the difference between studies may be attributed to differences in the EQ-5D version and value set used. Rodriguez Santana and colleagues [18] used the EQ-5D-5L with utility calculated using the England value set (the specific version of the England value set was not reported); Carlozzi and colleagues [16] did not report which version of the EQ-5D instrument or which value set was used. Rodriguez Santana and colleagues used proxy completion for patients with a severe cognitive deficit (the mean for all respondents is reported earlier in this paragraph); Carlozzi and colleagues [16] did not report use of proxy completion. The use of proxy completion may also explain some of the difference in utility estimates, as the proxy utility value for advanced-stage disease was much lower than the patient-reported value (0.13 versus 0.42, respectively).

**Fig. 2:**
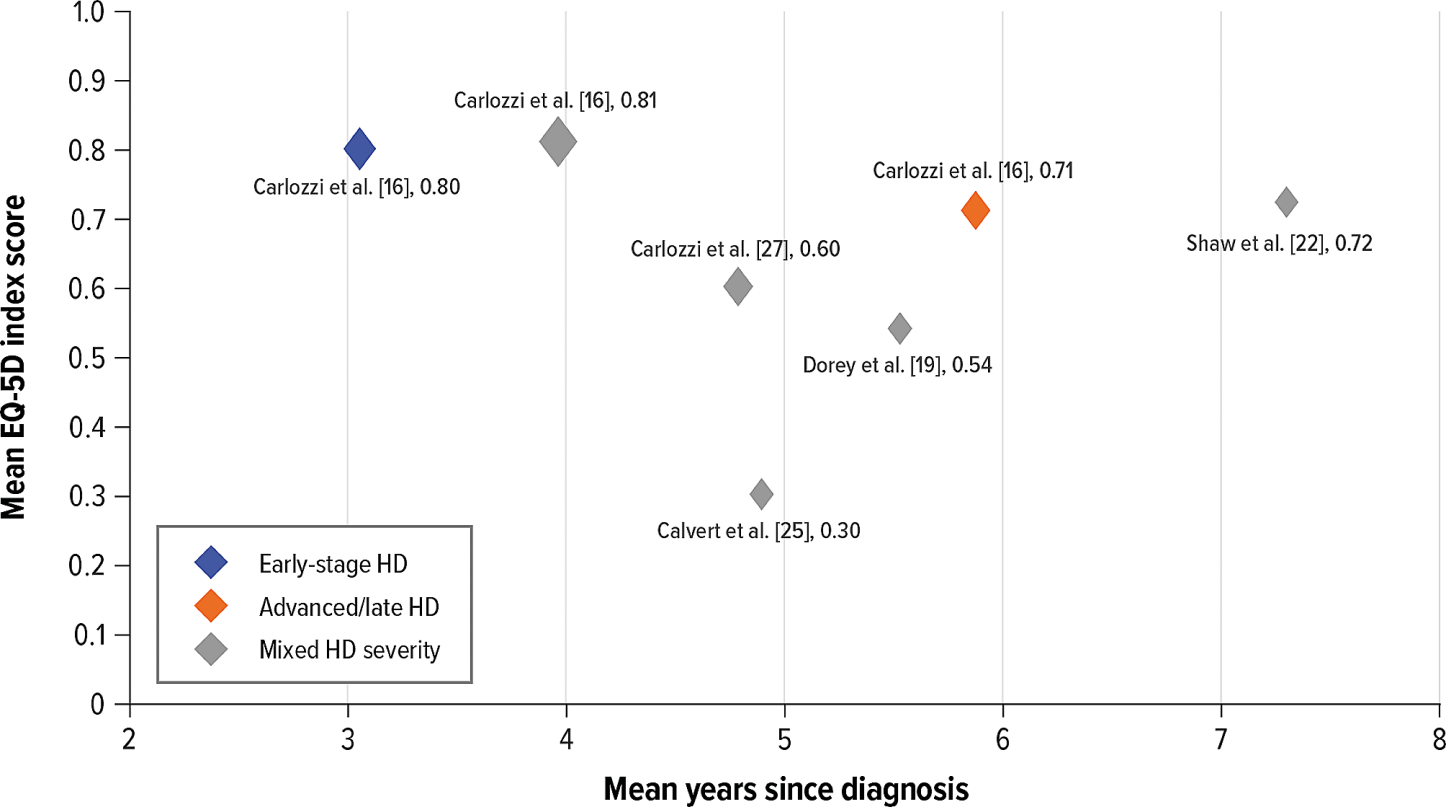
Comparison of published EQ-5D utility scores and mean time since diagnosis for patients with HD HD = Huntington’s disease; EQ-5D = EuroQol 5-dimension

A visual comparison of mean EQ-5D index utility scores across studies in patients with differing numbers of years since HD diagnosis does not reveal any obvious trend between time since HD diagnosis and mean utility value (Fig. 2). For example, in a US cross-sectional survey of patients with a mean 4.8 years since diagnosis, the mean (SD) EQ-5D index score was estimated to be 0.6 (0.3) [27]. In contrast, a Canadian cross-sectional survey [22] of patients with a substantially longer average number of years since diagnosis (9.9 years) estimated the mean (SD) EQ-5D index score to be higher (0.72 [0.24]) than that in the US survey. In both studies, HD diagnosis and time since diagnosis were self-reported. Some of the difference in utility estimates may be explained by the fact that the Canadian study [22] estimated EQ-5D utility index from SF-36 data using the mapping algorithm by Rowen et al. [29], which has been reported to overpredict utility for more severe health states. However, a regression analysis of SF-6D data by Hawton and colleagues [23] found no significant relationship between utility and time since diagnosis. The authors acknowledged some individuals are diagnosed in the premanifest stage because of family history of the condition and early predictive testing and may live for decades after diagnosis without any clinical expression, while others may be diagnosed much later at the point of significant functioning loss. Such heterogeneity in the timing of diagnosis might have made it difficult to interpret the relationship between years since diagnosis and severity levels or health statuses of patients with HD.

Two studies reported SF-6D estimates [18, 23]. In a longitudinal, observational study of patients with clinically diagnosed HD in 12 European countries, Hawton and colleagues [23] reported that SF-6D utility estimates declined with disease stage: TFC 13 − 11: 0.767 through TFC 2 − 0: 0.575. Utility estimates were not markedly lower than general population normative data for older people. For example, SF-6D utility was 0.68 compared with 0.73 for the general population in people aged 75–79 years. Rodriguez Santana et al. also reported that a decline in SF-6D utility corresponded with progression of disease stage, with early stage (TFC 13 − 7) yielding 0.61, through advanced stage (TFC 3 − 0) characterized by significant and total dependence on external care [28] experiences the most severe symptoms and require assistance in all activities of daily living, yielding 0.50. The decline in SF-6D utility with worsening TFC (0.61 to 0.50 from early to advanced stage) was smaller than the change in EQ-5D utility measured in the same study (0.72 to 0.37 from early manifest HD to advanced stage HD). A larger difference in EQ-5D utility between patients with HD and the general population was observed by Exuzides and colleagues [21] (0.66 and 0.81, respectively), suggesting that SF-6D may be less sensitive to the changes in HRQOL with HD progression than EQ-5D.

One vignette valuation study was identified [17]. In this study, health state descriptions (vignettes) were developed for different severities of HD chorea (with other manifestations remaining constant), and the health states were valued by general population participants using time trade-off methods. The mean (SD) utility values declined as chorea severity increased (mild: 0.64 [0.41]; mild/moderate: 0.48 [0.47]; moderate/severe: 0.26 [0.50]; severe: 0.07 [0.52]).

**Fig. 3:**
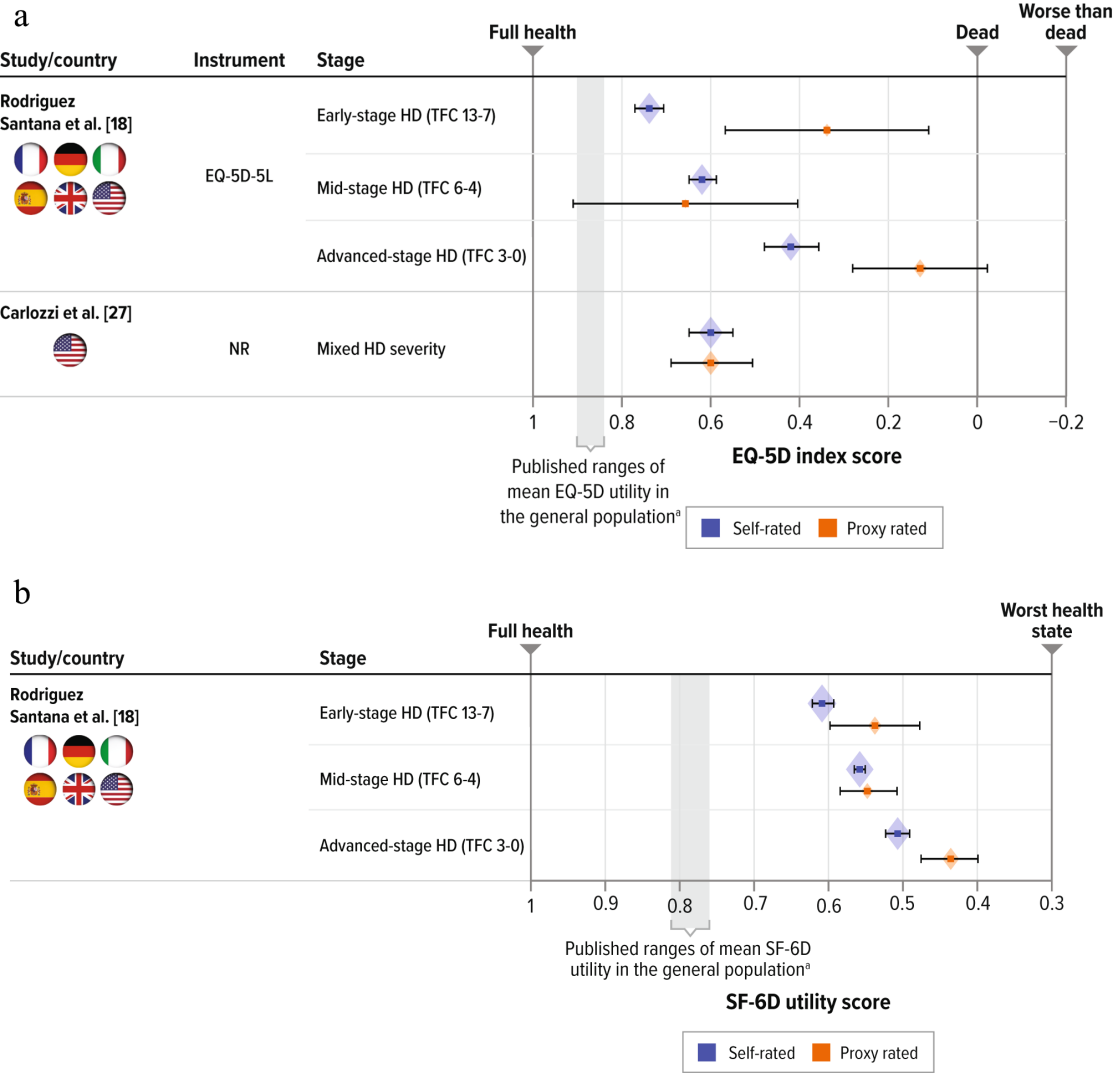
Comparison of published utility scores for patients with HD by rater type. **a.** Utility Scores Measured by EQ-5D. **b.** Utility Scores Measured by SF-6D HD = Huntington’s disease; EQ-5D = EuroQol 5-dimension; EQ-5D-5L = EuroQol 5-dimension 5-level; SF-6D = short-form 6-dimension; TFC = total functional capacity Note: Error bars represent 95% confidence intervals derived from reported standard deviation and sample size (size of diamond represents the sample size). Country refers to location of residence of study participants ^a^ Range for published mean EQ-5D and SF-6D utility for general US population of ages 35 to 64 years [49].

Among the 11 studies reviewed, 2 reported utility values assessed by proxy respondents on behalf of patients (Fig. 3). In a cross-sectional survey by Carlozzi et al. [27], the patient- and proxy-reported mean EQ-5D utility index scores were identical (both 0.6), although the mean proxy-reported TFC score was substantially lower than the mean patient-reported score (5.4 vs. 6.8), suggesting a disconnect in functioning capabilities by perspective. Similarly, in a cross-sectional survey by Rodriguez Santana et al. [18], proxy respondents reported similar mean EQ-5D scores as patients with mid-stage HD (0.62 [patient reported] vs. 0.66 [proxy reported]). However, large differences were reported in patients with advanced HD, where the mean (SD) score for patients was 0.42 (0.27) compared with 0.13 (0.31) for proxies. Part of this difference may be attributed to the study design in that proxy rating was used only for participants with a severe cognitive deficit.

## Discussion

**Fig. 4:**
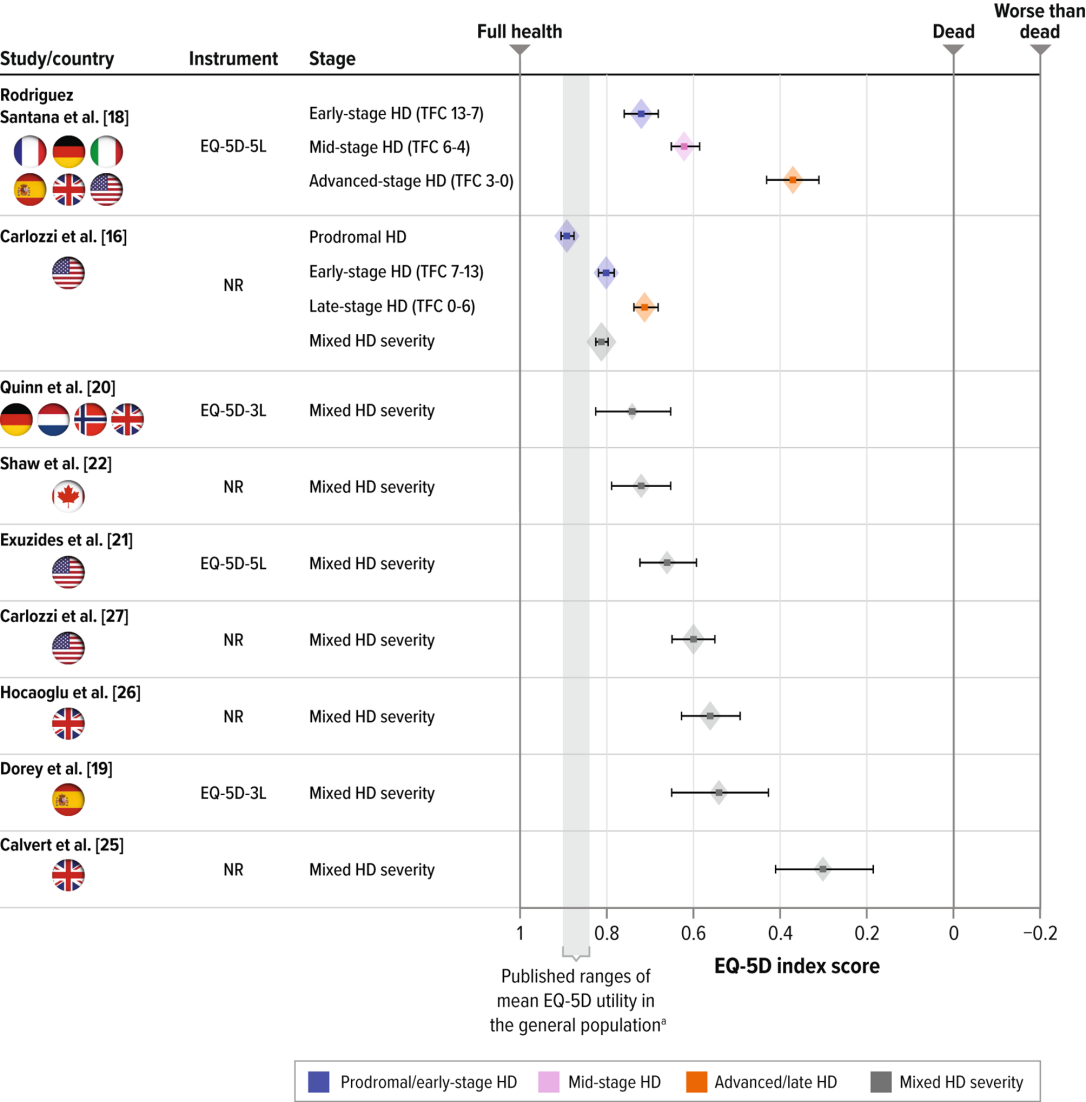
Comparison of published EQ-5D index scores for patients with HD HD = Huntington’s disease; EQ-5D = EuroQol 5-dimension; EQ-5D-5L = EuroQol 5-dimension 5-level; EQ-5D-5L = EuroQol 5-dimension 3-level; NR = not reported; TFC = total functional capacity Note: Error bars represent 95% confidence intervals derived from reported standard deviation and sample size (size of diamond represents the sample size). Country refers to location of residence of study participants ^a^ Range for published mean EQ-5D utility for the general US population ages 35 to 64 years [49]

This systematic literature review confirmed that published HSU scores estimated via generic preference-based utility measures (EQ-5D and SF-6D) are available for patients with HD. In studies that reported utility estimates for different stages of HD (such as early-, mid-, and late-stage HD), mean utility scores were numerically lower in patients with more advanced stages of manifest HD. Figure 4 illustrates the EQ-5D index scores associated with different severity levels of HD. While mean utility values for patients with prodromal or early manifest HD were between 0.89 and 0.72, mean utility values for patients with late-manifest HD ranged from 0.71 to 0.37. This variability in estimates among studies is problematic for researchers developing formal value assessments based on QALYs, as the different utility estimates would be expected to result in very different QALY estimates. While some of the variability may be explained (e.g., by differences in the definition of the severity categories, in the EQ-5D version and value set used, and/or in the use of proxy respondents for some patients in some studies), it is unclear which estimates are most appropriate to use in cost-effectiveness analyses.

The high mean EQ-5D index score in patients with early manifest HD reported in Carlozzi et al. [16] may indicate a challenge associated with using EQ-5D to quantify the impact of early manifest HD, as the estimate is close to published data for the general population matched by age and sex (0.81) [21]. However, Rodriguez Santana et al. [18] reported a somewhat lower EQ-5D index for early-stage disease (0.72). People in the early manifest stages of HD are likely to have cognitive, behavioral, and motor impairment, which may significantly impact their day-to-day functioning, QOL, and ability to stay independent [16, 30–32]. Additionally, those who have manifest HD may have significant cognitive impairment and not be fully aware of or able to accurately reflect on their own health status, and proxy reporting may be appropriate in such cases [33].

**Fig. 5:**
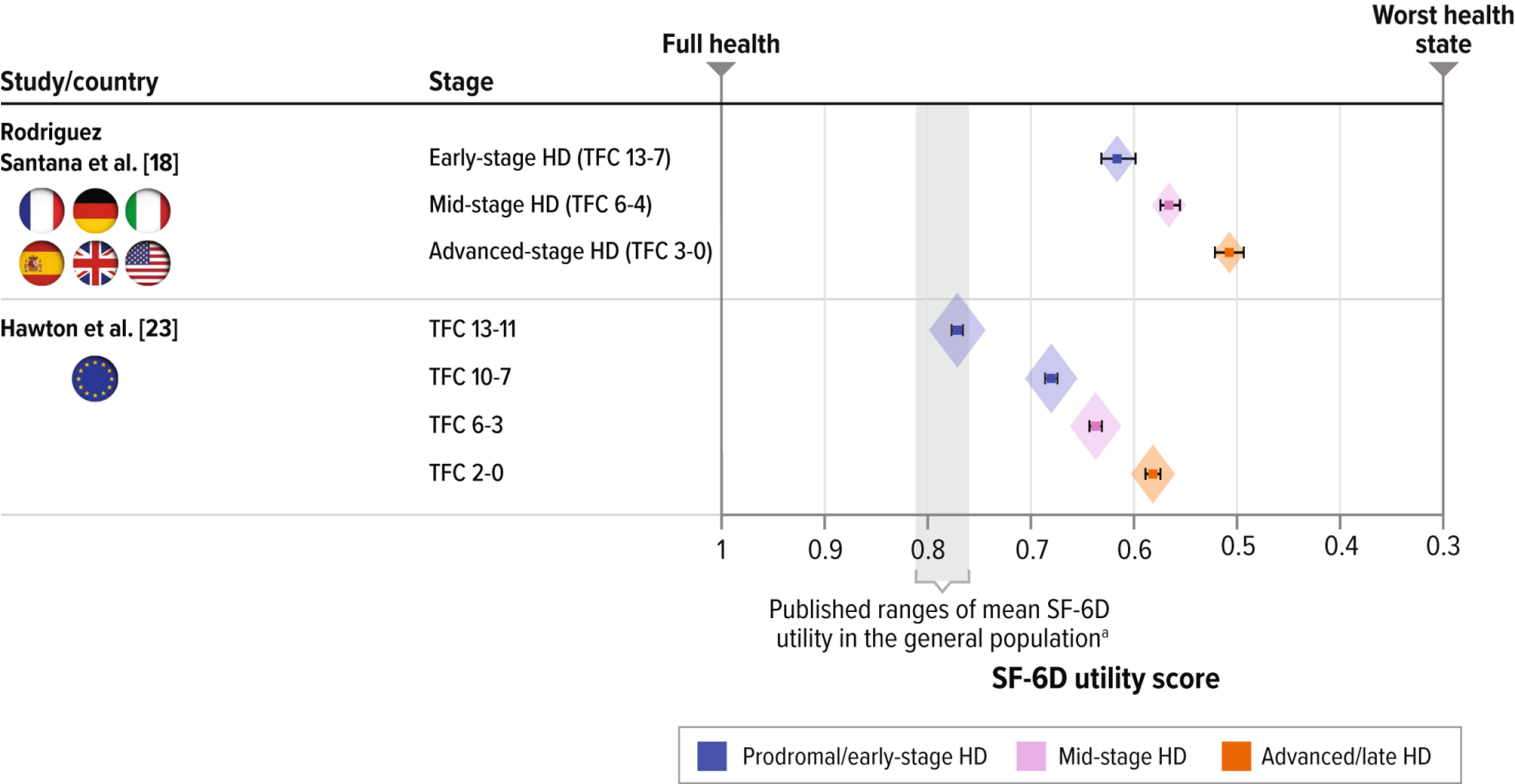
Comparison of published SF-6D utility scores for patients with HD HD = Huntington’s disease; SF-6D = short-form 6-dimension; TFC = total functional capacity Note: Error bars represent 95% confidence intervals derived from reported standard deviation and sample size (size of diamond represents the sample size). Country refers to location of residence of study participants ^a^ Range for published mean SF-6D utility for general US population of ages 35 to 64 years [49]

Our review confirmed that SF-6D–based utility estimates decreased with worsening HD severity in both studies (Fig. 5) [18, 23]. Hawton et al. [23] identified strong relationships between behavioral symptoms (such as sad mood, low self-esteem, guilt, disruptive or aggressive behavior, obsessions, hallucinations, irritable behavior) as well as functional independence and SF-6D utility values, but little relationship between cognitive symptoms or motor symptoms and SF-6D scores. This highlights a potential limitation of using SF-6D for capturing the effects of cognitive and motor impairments on the HSU in HD. Furthermore, the change in SF-6D utility with TFC decline was substantially smaller than the change in EQ-5D utility measured in Rodriguez Santana et al. [18]. SF-6D seems to be less sensitive than EQ-5D to the changes in HRQOL with HD symptom progression.

Cognitive symptoms are a key driver of disability and functioning deficits in HD, and often results in loss of work and independent functioning [32, 34]. Therefore, it is unclear whether EQ-5D is appropriate for measuring HRQOL impacts associated with cognitive impairment in HD. The EuroQol Group (which administers the EQ-5D instrument) has recognized that several studies have identified cognition as an area for which the psychometric properties of the EQ-5D may be weak [e.g. 35–37]; further research is ongoing. McGrath et al. [38] concluded that the EQ-5D descriptive system seemed to have poorer alignment with the impacts of Alzheimer’s Disease than other generic utility measures, and this also may be the case for the impacts of cognitive impairment in HD. Addition of cognition bolt-on dimensions has been shown to affect preference values for EQ-5D health states [39, 40], which suggests that preferences for cognition health states could be better measured than by the EQ-5D alone.

No value set (providing utility values for all the possible health states) is currently available for the EQ-5D including the cognition bolt-on, so this cannot be used to calculate QALYs. Cognitive changes often appear in the prodromal stage, prior to motor onset (diagnostic confidence level < 4) [41, 42] and can include a decline in executive function (including working memory, flexible thinking, and self-control), memory problems, and difficulty concentrating [34]. Further research into the appropriateness of the EQ-5D and whether other generic utility measures such as the Health Utilities Index, 15 Dimensions, or EQ Health and Wellbeing (EQ-HWB) instrument, which include specific dimensions for cognition and mental function, would be valuable. Vignette valuation studies or development of a new disease-specific utility measures could provide a possible solution given the lack of alternatives [43]; however, their use comes with recognized limitations [44, 45]. The EQ-HWB measure (currently under development) may also address this gap for measuring cognition [46].

## Conclusions

Health utility in HD has been studied using the EQ-5D, SF-6D, and vignette valuation. Studies identified in this systematic literature review showed high variability in published utility estimates, indicating substantial uncertainty in the existing data. It is unclear which reported utility values should be utilized in value assessments of new treatments for HD. There is some evidence to suggest that SF-6D may be less sensitive than EQ-5D to measuring HRQOL impairment in HD.

Further research is needed to better understand preferences and valuation in all stages and symptom clusters of HD, the impact of symptoms and manifestations of HD on patients, and the degree to which current generic utility measures are sensitive in capturing the impact of symptoms and manifestations of HD on patients’ HRQOL. Challenges in measuring health utility in patients with HD during functional decline may be due to the fact that cognitive impairment and behavioral changes are major symptoms that manifest in the early phase in the disease course. These symptoms are generally not captured by generic instruments such as the EQ-5D and SF-6D. Appropriateness of generic utility measures in HD should be explored.

### Electronic supplementary material

Below is the link to the electronic supplementary material.

Supplementary Material 1

## Data Availability

Data sharing is not applicable to this article as no datasets were generated or analyzed during the current study.

## Abbreviations

EQ-5D-5L: EuroQol 5-Dimension 5-Level
EQ-5D-3L: EuroQol 5-Dimension 3-Level
EQ-5D: EuroQol 5-Dimension
EQ-HWB: EQ Health and Wellbeing
H-QoL-I: Huntington Quality of Life Instrument
HD-PRO-TRIAD: Huntington’s Disease-specific patient-reported outcome instrument of the HD symptom triad
HD: Huntington’s Disease
HDQLIFE: Huntington Disease Health-Related Quality of Life
HDQoL: Huntington’s Disease health-related Quality of Life
HRQOL: Health-related quality-of-life
HSU: Health state utility
ISPOR: Professional Society for Health Economics and Outcomes Research
MeSH: Medical Subject Headings
PRISMA: Preferred Reporting Items for Systematic Reviews and Meta-Analyses
QALY: Quality-adjusted life-year
QOL: Quality of life
SD: Standard deviation
SF-36: 36-Item Short-Form Survey
SF-6D: Short-Form 6-Dimension
TFC: Total Functional Capacity
TTO: Time trade-off
VAS: Visual analogue scale
