# Supplementary material for: Health state utility estimates for value assessments of novel treatments in Huntington’s disease: a systematic literature review
Source: Health Qual Life Outcomes. 2024 Apr 16;22:33. doi: 10.1186/s12955-024-02242-1 (PMC11020898; doi:10.1186/s12955-024-02242-1)
Supplement: Supplementary file 1 — Supplementary Material 1 [file 12955_2024_2242_MOESM1_ESM.pdf]

Health State Utility Estimates for Value Assessments of Novel Treatments in Huntington's Disease: A Systematic Literature Review

Ruta Sawant, PhD, Health Economics and Outcomes Research, Sage Therapeutics, Inc., 215 First Street, Cambridge, MA, United States
Kyle Paret, PhD, Health Economics, RTI Health Solutions, 3040 East Cornwallis Road, Research Triangle Park, NC, United States
Jennifer Petrillo, PhD, Health Economics and Outcomes Research, Sage Therapeutics, Inc., 215 First Street, Cambridge, MA, United States
Aaron Koenig, MD, Medical Science, Sage Therapeutics, Inc., 215 First Street, Cambridge, MA, United States
Sorrel Wolowacz, PhD, Health Economics, RTI Health Solutions, The Pavilion, Towers Business Park, Wilmslow Road, Didsbury, Manchester, United Kingdom
Naoko Ronquest, PhD, Health Economics, RTI Health Solutions, 3040 East Cornwallis Road, Research Triangle Park, NC, United States
Hugh Rickards, MD, Institute of Clinical Sciences, College of Medical and Dental Sciences, University of Birmingham, 32-34 Colmore Circus Queensway, Birmingham, United Kingdom

Corresponding Author:
Ruta Sawant
Sage Therapeutics, Inc.
215 First Street
Cambridge, MA 02142
Email: ruta.sawant@sagerx.com

Health and Quality of Life Outcomes

Supplementary Information

Contents: List of Criteria for the Inclusion and Exclusion of Studies During Level 1 and Level 2 Screening Process; PubMed Literature Search Strategy

Table S1. List of Criteria for the Inclusion and Exclusion of Studies During Level 1 and Level 2 Screening Process

Criterion	Included	Excluded
Population	▪ Patients with HD	▪ Patients without HD
Interventions and comparators	▪ All	▪ None
Outcomes	▪ Utility outcomes based on PRO instruments such as: EQ-5D, SF-6D, HUI, 15D, PROMIS Preference Score<br>▪ Utility outcomes mapped from another generic HRQoL PRO measure (e.g., SF-12, SF-36) or disease-specific measures<br>▪ Utility values elicited from vignette valuation or time trade-off	▪ Studies that report only clinical efficacy and safety data<br>▪ Studies that did not report utility outcomes
Study design	▪ Utility studies (including studies where utility weights were mapped from other instruments, such as disease-specific patient-reported outcome measures)<br>▪ Prospective studies reporting utility data (observational studies, clinical trials)<br>▪ Systematic reviews of economic analyses or utility<br>▪ Economic analyses (cost-effectiveness or cost-utility analyses)	▪ Commentaries and letters (publication type)<br>▪ Consensus reports<br>▪ News articles<br>▪ Nonsystematic reviews<br>▪ Commentaries and letters (publication type)<br>▪ Editorials<br>▪ Genetic or pathological studies<br>▪ Animal studies
Language	▪ English reports	▪ Non-English reports
Date	▪ 1 January 2011 to 30 December 2022	▪ Before 1 January 2011

HD = Huntington's disease; HRQoL = health-related quality of life; HUI = Health Utilities Index; PRO = patient-reported outcome; PROMIS = Patient-Reported Outcomes Measurement Information System; SF-12 = 12-Item Short-Form Survey; SF-36 = 36-Item Short-Form Survey; SF-6D = short-form 6-dimension.

Note: If it was unclear whether a study met any criterion during the level 1 screening process, the study was progressed to full-text screening to confirm its inclusion in the review.

Table S2. PubMed Literature Search Strategy

Search Number	Search Terms	Hits
**Population**		
#1	"Huntington Disease"[Mesh] OR "Huntington Disease"[Title/Abstract] OR "Huntington's Disease"[Title/Abstract] OR "Huntington Chorea"[Title/Abstract] OR "Huntington's Chorea"[Title/Abstract] OR "chorea Huntington"[Title/Abstract] OR "chorea major"[Title/Abstract] OR "chronic progressive chorea"[Title/Abstract] OR "hereditary chorea"[Title/Abstract]	20,883
**Study Type: Utilities and Quality of Life**		
#2	"health utility"[Text Word] OR "health utilities"[Text Word] OR "standard gamble"[Text Word] OR "time trade off"[Text Word] OR "time trade-off"[Text Word] OR "tto"[Text Word] OR "euroqol"[Text Word] OR "euroqol 5d"[Text Word] OR "eq5d*"[Text Word] OR "eq 5d"[Text Word] OR "eq-5d"[Text Word] OR "assessment of quality of life"[Text Word] OR "aqol"[Text Word] OR "quality of well being"[Text Word] OR "quality of well-being"[Text Word] OR "qwb"[Text Word] OR "qwb-sa"[Text Word] OR "quality of well being self-administered"[Text Word] OR "quality of well-being self-administered"[Text Word] OR "quality of well-being-self-administered"[Text Word] OR "quality of well being-self administered"[Text Word] OR "15d"[Text Word] OR "15-dimensional"[Text Word] OR "15 dimensional"[Text Word] OR "fifteen-dimensional"[Text Word] OR "fifteen dimensional"[Text Word] OR "health utility index"[Text Word] OR "health utilities index"[Text Word] OR ("health"[Text Word] AND "utilit*"[Text Word] AND "index"[Text Word]) OR "sf-6d"[Text Word] OR "sf6"[Text Word] OR "sf 6"[Text Word] OR "short form 6"[Text Word] OR "shortform 6"[Text Word] OR "sf six"[Text Word] OR "sfsix"[Text Word] OR "shortform six"[Text Word] OR "short form six"[Text Word] OR "qaly"[Text Word] OR "Quality-Adjusted Life Years"[Mesh] OR "quality adjusted life year"[Text Word] OR "quality adjusted life years"[Text Word] OR "quality adjusted life-year"[Text Word] OR "quality-adjusted life-year"[Text Word] OR "quality-adjusted life years"[Text Word] OR "quality adjusted life-years"[Text Word] OR "quality-adjusted life-years"[Text Word] OR "daly"[Text Word] OR "dalys"[Text Word] OR "disability adjusted life year"[Text Word] OR "disability adjusted life years"[Text Word] OR "willingness to pay"[Text Word] OR ("utilit*"[Text Word] AND "score*"[Text Word]) OR ("utilit*"[Text Word] AND "weight*"[Text Word]) OR disutility*[Text Word] OR (utilit*[Text Word] AND (valu*[Text Word] OR measur*[Text Word] OR health[Text Word] OR life[Text Word] OR estimat*[Text Word] OR elicit*[Text Word] OR disease[Text Word] OR score*[Text Word] OR weight[Text	306,503

Search Number	Search Terms	Hits
	Word])) OR "short form six"[Text Word] OR sf36[Text Word] OR "sf 36"[Text Word] OR "short form 36"[Text Word] OR "shortform 36"[Text Word] OR "short form36"[Text Word] OR "shortform36"[Text Word] OR "sf thirtysix"[Text Word] OR "sfthirtysix"[Text Word] OR "sfthirty six"[Text Word] OR "sf thirty six"[Text Word] OR "shortform thirtysix"[Text Word] OR "shortform thirty six"[Text Word] OR "short form thirtysix"[Text Word] OR "short form thirty six"[Text Word] OR (health*[Text Word] AND year*[Text Word] AND equivalent*[Text Word]) OR "Nottingham Health profile"[Text Word] OR "Sickness Impact Profile"[Mesh] OR "sickness impact profile"[Text Word] OR "health utilities index"[Text Word] OR HUI[Text Word] OR "Caregiver Burden"[Mesh] OR "caregiver burden"[Text Word] OR "health related quality of life"[Text Word] OR "PROMIS"[Text Word] OR "preference score"[Text Word]	
#3	#1 AND #2	333
**Exclusion**		
#4	– "Animals"[Mesh] NOT "Humans"[Mesh]	
#5	– "Comment"[Publication Type] OR "Letter"[Publication Type] OR "Editorial"[Publication Type] OR "Case reports"[Publication type] OR "case stud*"[Title] OR "case report*"[Title] OR "case series"[Title] OR "case histor*"[Title]	
**Total**		
#6	#3 NOT (#4 OR #5)	288
#7	#6 AND ("2012/01/01"[Date - Publication]: "2022/12/31"[Date - Publication])	201
#8	#7 AND English[Language]	**200**
